# Morphological and Mechanical Characterization of DNA SAMs Combining Nanolithography with AFM and Optical Methods

**DOI:** 10.3390/ma13132888

**Published:** 2020-06-27

**Authors:** Giulia Pinto, Paolo Canepa, Claudio Canale, Maurizio Canepa, Ornella Cavalleri

**Affiliations:** 1 Department of Physics, University of Genova, via Dodecaneso 33, 16146 Genova, Italy; pinto@fisica.unige.it (G.P.); paolo.canepa@edu.unige.it (P.C.); canepa@fisica.unige.it (M.C.); 2 OPTMATLAB, Department of Physics, University of Genova, via Dodecaneso 33, 16146 Genova, Italy

**Keywords:** AFM, Spectroscopic Ellipsometry, DNA, self-assembled monolayers, ionic strength, molecular absorption

## Abstract

The morphological and mechanical properties of thiolated ssDNA films self-assembled at different ionic strength on flat gold surfaces have been investigated using Atomic Force Microscopy (AFM). AFM nanoshaving experiments, performed in hard tapping mode, allowed selectively removing molecules from micro-sized regions. To image the shaved areas, in addition to the soft contact mode, we explored the use of the Quantitative Imaging (QI) mode. QI is a less perturbative imaging mode that allows obtaining quantitative information on both sample topography and mechanical properties. AFM analysis showed that DNA SAMs assembled at high ionic strength are thicker and less deformable than films prepared at low ionic strength. In the case of thicker films, the difference between film and substrate Young’s moduli could be assessed from the analysis of QI data. The AFM finding of thicker and denser films was confirmed by X-Ray Photoelectron Spectroscopy (XPS) and Spectroscopic Ellipsometry (SE) analysis. SE data allowed detecting the DNA UV absorption on dense monomolecular films. Moreover, feeding the SE analysis with the thickness data obtained by AFM, we could estimate the refractive index of dense DNA films.

## 1. Introduction

Nucleic acid microarrays are largely employed in biosensing: the parallel detection of DNA or RNA hybridization allows for the simultaneous multiple detection of biomarkers [1,2]. The key feature of these devices is the highly specific biorecognition deriving from Watson–Crick base pairing that can be exploited for the selective detection of different analytes from oligonucleotides up to cells [3].

Hybridization can be exploited for biosensing on a two-fold level. It can be used for the direct recognition of specific target sequences, with applications in molecular biology and molecular diagnostics, since particular diseases can be identified based on the identification of specific nucleic acid sequences. Examples are the detection of methicillin-resistant Staphylococcus aureus (MRSA) [4], mi-RNA sequences related to tumor or cardiovascular diseases [5,6], or coronavirus-related sequences [7,8]. In this respect, in response to the pandemic COVID-19 emergency, the selective recognition of SARS-CoV-2 sequences has been recently demonstrated using a thermoplasmonic approach based on DNA hybridization [9]. On the other hand, in a supramolecular scheme, hybridization can be exploited for the immobilization of selective and functional sensing molecules: in DNA-directed molecular anchoring, complementary DNA bearing the sensing molecule can hybridize with immobilized DNA. In this way, the sensing molecules can be anchored to the surface, but they are uncoupled from it by the DNA layer, and therefore maintain their functionality and selectivity toward specific molecular biomarkers [10,11,12]. The detection of molecular binding events often involves the use of fluorescent labels [13,14]. Our aim is to develop a label-free detection scheme, by combining Atomic Force Microscopy (AFM) nanolithography and Spectroscopic Ellipsometry (SE).

The design of a robust and reliable DNA biosensor passes through the careful characterization of the DNA monolayer. Compared to the self-assembly of simpler molecules such as alkanethiols, the chemisorption of thiolated DNA strands on gold is a more complex process, since electrostatic interactions as well as conformation-dependent steric hindrance play a role in the process. This explains the role of the ionic strength in DNA conformation [15,16] and self-assembly [17,18,19,20], and motivates, for instance, the use of spacer molecules to promote the molecular organization of the DNA strands [17,21,22]. Different experimental approaches have been employed to investigate DNA self-assembly on gold, from electrochemical methods [18,22,23,24], to optical [20,25,26], infrared [22,27,28], and X-ray [17,28,29] spectroscopy. An aspect that has not been addressed so far is related to the mechanical properties of DNA Self-Assembled Monolayers (SAMs). The evaluation of the mechanical properties of ultrathin films such as SAMs is a challenging task, since a high sensitivity is required to disentangle the film contribution from the substrate one. Indeed, the evaluation of the mechanical properties of thin and ultrathin organic layers is an important issue for the development and optimization of biosensors, since it has been demonstrated that the interaction between substrates and biosamples, in particular cells, is strongly influenced by the sample stiffness [30]. Here, we employ scanning probe microscopy to investigate the morphological and mechanical properties of DNA SAMs, showing that the method is sensitive enough to discriminate between DNA layers self-assembled at different ionic strengths. Scanning probe nanolithography methods are versatile tools that allow for a precision patterning of ultrathin organic monolayers [31,32]. Since the first reports by Liu and coworkers [33,34], scanning probe nanolithography has been successfully employed to produce patterned organic/inorganic interfaces for the precise immobilization of nano-objects (from nanoparticles to biomolecules) [35] as well as to develop sensing platforms that are able to detect molecular recognition events [36,37,38].

In parallel to patterning, nanoshaving experiments can be advantageously employed to measure the thickness of molecular layers down to the subnanometer level, thus allowing monitoring film thickness changes following supramolecular multi-step assembly. At variance with previous reports that used the hard contact mode to displace molecules [34,39,40,41,42], we explore the use of the hard tapping mode to move the AFM tip as a shaver that selectively displaces molecules from the surface [43].

In addition to the more commonly used soft contact mode, imaging of the shaved areas was performed in Quantitative Imaging (QI) mode. QI is a force-distance curve-based microscopy mode that allows acquiring a height image and a map of different sample properties, simultaneously, with nanometer resolution. In particular, the analysis of the force curves allows evaluating local mechanical parameters such as the adhesion and the Young’s modulus [44].

To correlate structural, chemical, and optical properties of the DNA SAMs and to investigate their dependence on the ionic strength of the self-assembly solution, we couple nanoshaving experiments with spectroscopic measurements. X-rays Photoelectron Spectroscopy (XPS) allowed to detect the relevant molecular signals and to correlate their intensities to the molecular coverage. Optical spectroscopy measurements in the UV-VIS range provided information on the optical properties of the DNA films. Spectroscopic Ellipsometry is a reliable, rapid, highly sensitive, and not-perturbative optical technique that measures the variation in polarization of a light beam upon reflection from a surface, providing information on the thickness and optical properties of thin and ultrathin films. For ultrathin films, the thickness and refractive index are correlated parameters. We exploit the thickness data obtained from AFM nanolithography measurements to disentangle this correlation [40,45]. Indeed, the cross-fertilization between the two methods (scanning probe microscopy and Spectroscopic Ellipsometry) can be advantageously employed to obtain quantitative and consistent data on thickness and refractive index of monomolecular films, once a proper optical model of the system is provided. Moreover, through the analysis of ellipsometry difference spectra, we can detect the spectral features of the DNA absorption at the monolayer level and its dependence on the ionic strength.

## 2. Materials and Methods

### 2.1. Materials and Chemicals

Thiolated 22-mers SH-(CH2)6-5′-TAATCGGCTCATACTCTGACTG-3′ (C_6_-ssDNA) were purchased from Biomers (Ulm, Germany) and used as received. Single-stranded oligonucleotides functionalized with a thiolated alkyl chain can self-assemble more easily and form more stable films than no-thiolated oligonucleotides thanks to the formation of the S-Au bond [17,26].

Tris[hydroxymethyl]amino-methane (Tris base) and ethylenediamine-tetraacetic acid (EDTA) were purchased from Sigma Aldrich (St. Louis, MO, USA). Sodium chloride (NaCl) was purchased from Merck (Darmstadt, Germany). Sulfuric acid (H_2_SO_4_) and 30% hydrogen peroxide (H_2_O_2_) were purchased from Carlo Erba (Val de Reuil, France). Milli-Q water from Millipore (resistivity ≥18 MΩ·cm) was used in all the experiments.

Experiments were performed using TE buffer (10 mM Tris, 1 mM EDTA and NaCl, pH adjusted at 7.2 using HCl (Fluka, Buchs, Switzerland)). Two NaCl concentrations were used, 1 mM NaCl (1 mM NaCl buffer) or 1 M NaCl (1 M NaCl buffer). The two salt concentrations (1 mM and 1M) were chosen as extreme values to evaluate the effect of salt. Below 1 mM NaCl, because of the very low density of deposited molecules, AFM, X-Ray Photoelectron Spectroscopy (XPS), and SE data lose their reliability due to the significant experimental uncertainties. On the other hand, based on previous reports [17,18,19], 1 M NaCl was chosen as a sufficiently high concentration to guarantee a full coverage molecular layer.

Flat gold substrates purchased from Arrandee (root mean square (RMS) roughness = 2.4 nm in 2 µm × 2 µm scan, (Werther/Westfalen, Germany) were used for ellipsometric and XPS measurements. Ultraflat gold substrates (RMS roughness = 0.3 nm in 2 µm × 2 µm scan) prepared according to Gupta et al. [46] (often called Ulman-type gold) were used for AFM measurements.

### 2.2. Samples Preparation

Arrandee substrates were cleaned with piranha solution (4:1 H_2_SO_4_:30% H_2_O_2_) for 3 min, thoroughly rinsed with Milli-Q water, and dried under a nitrogen stream (CAUTION—Piranha should be handled with extreme care: it is extremely oxidizing, reacts violently with organics, and should only be stored in loosely tightened containers to avoid pressure buildup). No cleaning procedures were necessary for ultraflat gold substrates, since the capping silicon slide was removed from the gold surface immediately prior to use.

Oligonucleotide self-assembly was carried out by keeping the clean gold substrates in 1 µM C_6_-ssDNA solution in TE buffer for 24 h at ambient temperature.

After C_6_-ssDNA self-assembly, samples were rinsed and characterized in situ by AFM in TE buffer. For each sample, at least four areas have been shaved to check for sample uniformity.

For SE measurements on ultrathin organic films, in order to emphasize the film contribution to the SE data, we followed a difference spectra analysis method that has been previously applied to organic monolayers [40,47,48]. Therefore, cleaned Arrandee samples were first characterized in situ in TE buffer, subsequently incubated in the C_6_-ssDNA solution for 24 h, and then rinsed and characterized by SE in situ in TE buffer.

After SE analysis, samples were dried and characterized by XPS acquiring survey and high-resolution spectra. For each sample, at least three regions were analyzed to check for sample uniformity.

### 2.3. Atomic Force Microscopy (AFM)

AFM experiments were carried out using a JPK NanoWizard IV microscope (Bruker, Billerica, MA, USA). Samples were analyzed in 1 mM or 1 M NaCl buffer depending on the solution used for the C_6_-ssDNA self-assembly. Gold-coated Si cantilevers (DNP-S10, Bruker) with an elastic constant of 0.24 N/m and a tip radius of curvature of 10 nm were used for both shaving and imaging operation mode. Nanoshaving experiments were performed by scanning a selected area (typically a few µm wide) in hard tapping mode (setting a very small oscillating amplitude, typically 0.01 nm, with a free oscillation amplitude of 70–80 nm) to selectively displace molecules and obtain an exposed gold region. After the shaving, an image with larger scan size was acquired in both soft contact and Quantitative Imaging mode. QI mode is an imaging mode based on force spectroscopy: through the acquisition of a large set of force–distance curves, it allows reconstructing the sample topography from the z position of the tip at a specific force load. Since the tip is withdrawn from the surface between each pixel, there are almost no lateral forces, and dragging is avoided. The combination of imaging and force spectroscopy provides quantitative data on mechanical properties of the sample in addition to the height measurements.

Data have been analyzed with Gwyddion (v2.55) and JPKSPM Data Processing software.

### 2.4. Spectroscopic Ellipsometry (SE)

Spectroscopic Ellipsometry measurements were performed using a rotating compensator instrument (M-2000, J.A. Woollam Co., Lincoln, NE, USA, 245–1700 nm) equipped with a 75 W Xe lamp. Spectra have been collected in situ using a commercial liquid cell (J.A. Woollam Co., 0.5 mL).

To emphasize the contribution of the ultrathin organic layer, we analyzed difference spectra, which were obtained as the difference between the spectra acquired after the film deposition and the spectra measured on the substrate just prior to molecular deposition [40,47,49].

## 3. Results and Discussion

**Figure 1:**
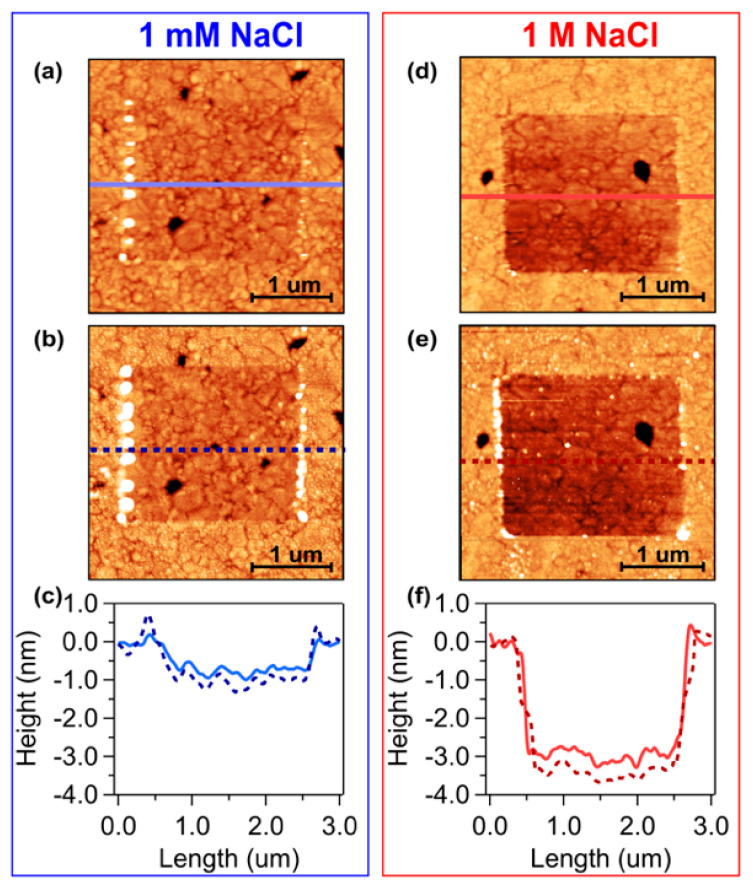
Shaving experiments on C_6_-ssDNA SAMs prepared in (**a–c**) 1 mM NaCl and (**d–f**) 1 M NaCl buffer. Height images of the shaved areas acquired in (**a,d**) contact mode and in (**b,e**) Quantitative Imaging (QI) mode (data scale: 9 nm). (**c,f**) z-profiles relative to contact images (light, continuous lines) and QI images (dark, dashed lines).

Figure 1 reports the results of two shaving experiments carried out on C_6_-ssDNA SAMs prepared in 1 mM NaCl buffer (a–c) and 1 M NaCl buffer (d–f), respectively. In a shaving experiment, molecules are selectively removed from specific regions by scanning over those regions with a high tip load. In particular, in the present experiments, C_6_-ssDNA molecules were removed by scanning over the selected region in hard tapping mode while QI mode, together with the standard soft contact mode, was exploited to image shaved areas. Both shaving and imaging were carried out in the same TE buffers used for the C_6_-ssDNA self-assembly.

The C_6_-ssDNA SAM thickness can be inferred from the analysis of height images through the evaluation of the height difference between the SAM region and the shaved region, i.e., the gold substrate. AFM height images acquired in soft contact mode on samples prepared in 1 mM NaCl buffer (1 mM NaCl C_6_-ssDNA SAMs) and 1 M NaCl buffer (1 M NaCl C_6_-ssDNA SAMs) are reported in Figure 1a,d, respectively. The corresponding QI height images are reported in Figure 1b,e, respectively. The darker squared regions correspond to the shaved areas where molecules have been removed. The SAM thickness can be evaluated from the profiles of the height images: Figure 1c,f show z profiles measured on the soft contact images (light, continuous curves) and on the QI images (dark, dashed curves), which were acquired on samples prepared in 1 mM (blue curves) and 1 M NaCl buffer (red curves), respectively.

The superposition of dashed and continuous curves indicates that the height estimation obtained in contact mode is slightly lower than the height value inferred from QI measurements. This difference can be attributed to the different operation mode: in contact mode, the continuous tip–sample interaction can result in drag forces that can perturb the monolayer, leading to a height underestimation. Therefore, it is important to minimize as much as possible the tip load. In QI mode, the intermittent tip–sample interaction avoids drag effects and results in a less perturbative operation mode. Therefore, a comparative analysis in soft contact and QI modes can increase the reliability of the SAM height estimation measured by AFM.

It is worth noting that the agreement between the z-profiles obtained in contact and QI modes is slightly better for films prepared at high ionic strength, indicating that these films are less vulnerable to the AFM tip perturbation. This finding is reasonably related to the fact that SAMs prepared at high salt concentration are thicker and therefore denser and more compact with respect to SAMs prepared at low salt concentration.

From the statistical analysis of z-profiles obtained analyzing different shaved areas in several samples, we obtained an average C_6_-ssDNA SAM thickness of (1.0 ± 0.3) nm for samples prepared in 1 mM NaCl buffer and of (3.3 ± 0.5) nm for samples prepared in 1 M NaCl buffer.

**Figure 2:**
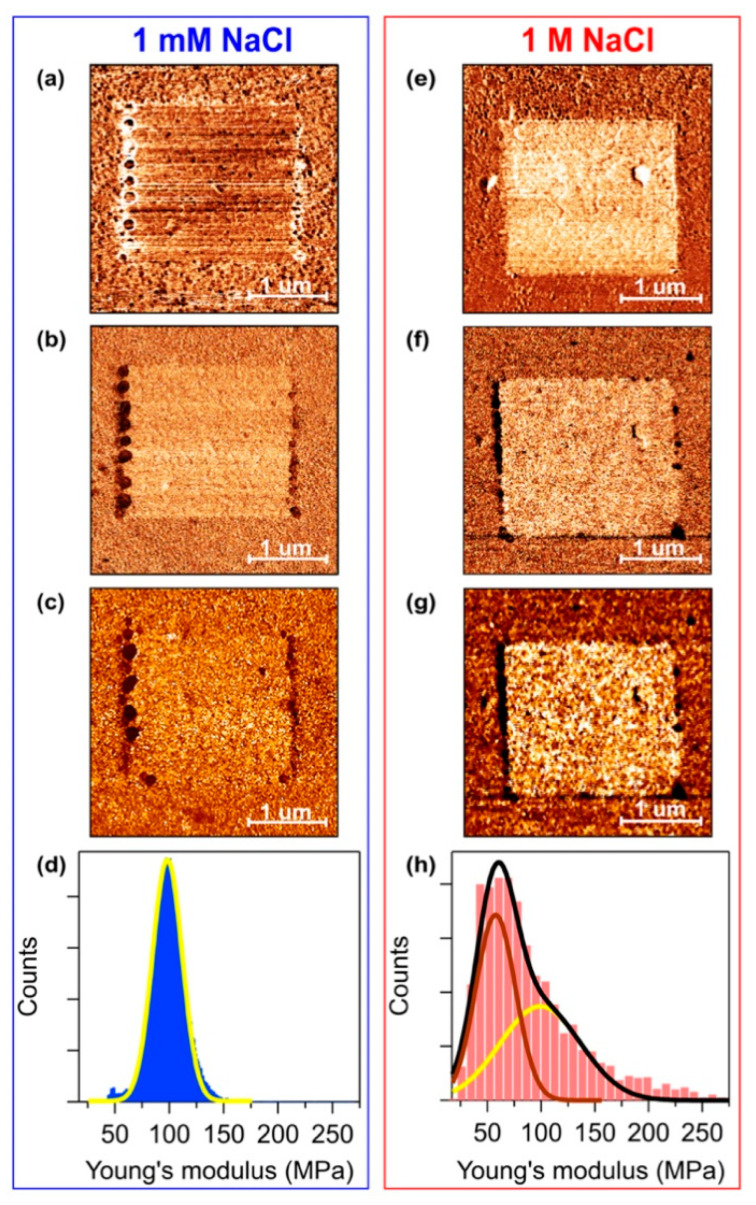
Shaving experiment on C_6_-ssDNA SAMs prepared in (**a–d**) 1 mM NaCl and (**e–h**) 1 M NaCl buffer. (**a,e**) Lateral deflection images acquired in contact mode (data scale: 20 mV). (**b,f**) Slope images acquired in QI mode (data scale: 250 N/m). (**c,g**) Young’s modulus images obtained from QI force curves (data scale: 150 MPa) and (**d,h**) related histograms.

In addition to film thickness, AFM measurements can provide information on the film mechanical properties. In Figure 2, we report the lateral deflection, slope, and Young’s modulus maps referred to the same shaving experiments analyzed in Figure 1.

Contact mode AFM can measure the cantilever lateral deflection, which gives information on the local friction between tip and sample surface. Therefore, we expect to measure a different lateral deflection when the tip scans over the C_6_-ssDNA SAM or over the gold substrate. The lateral deflection images reported in Figure 2 show that the difference in lateral deflection is very low in case of 1 mM NaCl C_6_-ssDNA SAMs (Figure 2a), while it is definitely higher for 1 M NaCl C_6_-ssDNA SAMs (Figure 2e). These results can be interpreted on the base of the height measurements reported above. C_6_-ssDNA SAMs prepared at low ionic strength have a subnanometer thickness: this indicates a low density molecular packing with almost lying down molecules. On such a layer, it is difficult to decouple the tribological properties of the film and of the gold substrate. Instead, at high salt concentration, a better organized and more compact layer is obtained: in this case, a definitely larger difference in lateral deflection is detected when the tip scans over the gold substrate (light square) or over the SAM.

The application of QI mode provides information not only on the sample morphology, but also on its mechanical properties. It is important to note that the evaluation of the mechanical properties of ultrathin films such as DNA SAMs is a challenging task, since it is difficult to uncouple the contribution of the substrate from that of the film. From the analysis of QI data, we could detect differences in the mechanical response of the films and of the gold substrate. In QI mode, the stiffness of the analyzed region can be inferred from slope maps obtained by calculating, for each acquired force–distance curve, the slope of a linear fit of the contact region close to the sample surface (see Figure S1 in Supplementary Materials). Assuming that the AFM probes have the same elastic constant and tip radius, the more rigid is the sample, the higher is the slope. On the other hand, on soft regions, the cantilever can deform the sample leading to a smaller deflection and therefore to a lower slope in the force–distance curve. In our experiments, the DNA region appears slightly darker (Figure 2b) and definitely darker (Figure 2f) for C_6_-ssDNA SAMs prepared at low and high ionic strength, respectively. Lower values of the slope over the film indicate that, as expected, the DNA film is softer than the gold substrate. We can also note that, as observed for lateral deflection images, the effect is faint at low salt concentration, while in the case of high ionic strength the difference between the stiffness of film and gold is definitely larger.

An additional analysis of QI data allows evaluating the Young’s modulus of the analyzed region (see Figure S1 in Supplementary Materials). Figure 2c,g show the 2D maps of the Young’s modulus values relative to the C_6_-ssDNA shaving experiments at low and high ionic strength, respectively. As expected, the gold substrate presents a definitely higher modulus with respect to the C_6_-ssDNA film in case of SAMs prepared in 1 M NaCl buffer, while at low salt concentration there is no significant difference between substrate and film. When present, the difference in the Young’s modulus values of C_6_-ssDNA film and gold substrate can be better appreciated by analyzing the histograms of the Young’s modulus maps reported in Figure 2d,h. The histogram in Figure 2d can be reproduced with a single Gaussian profile centered at 100 MPa, which means that in the case of SAMs deposited at low ionic strength, no significant difference can be detected between the Young’s modulus of the bare and SAM covered substrate. Conversely, at high ionic strength, two Gaussian profiles must be used to reproduce the histogram envelope (Figure 2h, black curve). A difference of about 40 MPa is detected between the two peaks, which correspond to the SAM (brown Gaussian profile, centered at 57 MPa) and to the substrate (yellow Gaussian profile, centered at 100 MPa). In agreement with the slope analysis results, the Young’s modulus values of the biofilm result, as expected, lower than those of the gold substrate.

**Figure 3:**
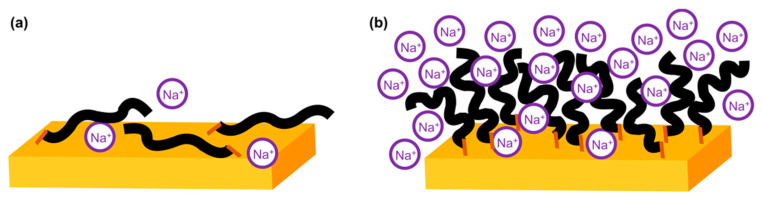
Sketches of C_6_-ssDNA SAMs immobilized in (**a**) 1 mM NaCl and (**b**) 1 M NaCl TE buffer.

Nanoshaving experiments indicate that the buffer ionic strength strongly influences the C_6_-ssDNA deposition. At low ionic strength, the highly charged DNA strands assume an elongated conformation and repel each other. A thin, low-density SAM of almost lying down molecules is formed. Conversely, at high ionic strength, the screening of the DNA charges leads to a more coiled strand conformation and reduces inter-strand repulsion. A thicker, higher coverage SAM formed by almost upright molecules is obtained in these conditions. This picture (Figure 3) is in agreement with the observation, for a set SAM molecular density, of elongated DNA strands at low ionic strength and mushroom-like strands at high ionic strength [50,51].

Further evidence of the higher film coverage at high salt concentration was obtained by XPS measurements (see Figures S2 and S3 in Supplementary Materials). High-resolution XPS spectra indicate the presence of all the relevant molecular signals, which can be deconvoluted to identify the different molecular groups present in the SAMs. The comparative XPS analysis of C_6_-ssDNA films prepared in high and low ionic strength buffers indicates the presence of the same molecular species in the two systems, but the intensity ratio between molecular and gold signals is almost three times higher in SAMs prepared using 1 M NaCl buffer. Even though the increase in the intensity ratio of the XPS signals cannot be directly translated into the increase of the layer thickness, the XPS finding is in good agreement with the AFM data, which indicates that SAMs prepared at high ionic strength are about three times thicker than SAMs assembled at low ionic strength.

We note that the ionic strength plays a similar role on the molecular density of DNA self-assembled on nanoparticles [52], which is a widely exploited system for biosensing purposes. However, self-assembly on nanoparticles is more complex than self-assembly on planar surfaces since additional parameters, such as the nanoparticle radius of curvature, come into play. 

The AFM/XPS results have been confirmed by SE analysis. Indeed, information on the film compactness could be inferred from SE data through the estimation of the film refractive index. As discussed below, the coupling between AFM and SE is particularly useful in the analysis of ultrathin layers, such as the DNA films.

**Figure 4:**
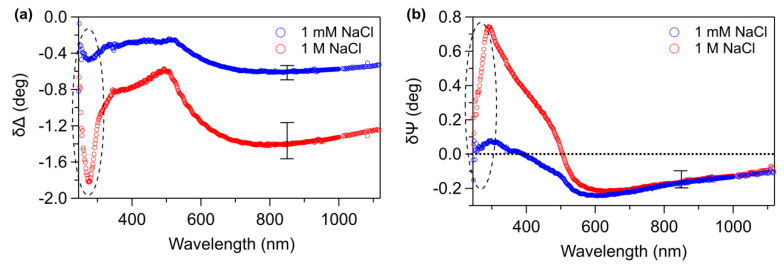
(**a**) δΔ and (**b**) δΨ spectra of 1 mM NaCl C_6_-ssDNA (blue curve) and 1 M NaCl C_6_-ssDNA (red curve). Error bars take into account the sample to sample variability. Dashed regions indicate fingerprint dips related to 260 nm DNA absorption.

Difference ellipsometric spectra of C_6_-ssDNA SAMs are reported in Figure 4. A remarkable spectral feature of the SE curves is the presence of a deep minimum around 270 nm in δΔ (Figure 4a) and a corresponding dip around 290 nm in δΨ (Figure 4b). These dips are well defined at high ionic strength (red curves), but they are present, even though weaker, also at low ionic strength (blue curves). Considering that the UV-Vis absorption of DNA in solution exhibits an absorption band located at 260 nm (confirmed by UV-Vis absorption spectroscopy, data not shown) and that transparent films present a very different behavior in this spectral region [53], we recognize these features as fingerprints of molecular DNA absorption. With a similar approach, we could previously identify molecular related UV-Vis absorptions by the analysis of difference SE spectra of biomolecular SAMs [49,54]. Recent papers focused on the optical properties of DNA thin solid films [55,56,57], but to the best of our knowledge, this is the first experimental observation in SE difference spectra of molecular absorption on DNA monolayers chemisorbed on gold.

δΔ and δΨ spectra exhibit also some typical properties of difference spectra of thiolate SAMs on gold [58]. In particular, δΔ spectra present a relative maximum (Figure 4a) around 500 nm, at the high reflectivity threshold of gold; in the same wavelength region, δΨ spectra (Figure 4b) show a well-defined transition to lower values with a minimum around 600 nm [59,60]. We previously associated the negative δΨ values in NIR region to an interface-related effect that characterizes the formation of organic films strongly anchored to the substrate [53,59].

SE data confirm that the ionic strength of the buffer plays a significant role in the SAM formation. As can be observed in Figure 4, |δΔ| values in the NIR (far from molecular resonances) and δΨ values in the near UV are definitely larger for C_6_-ssDNA SAMs deposited in 1 M NaCl buffer with respect to SAMs deposited in 1 mM NaCl buffer. According to previous reports [45,58], these features are associated to the film optical thickness, a quantity depending on refractive index and thickness, that is higher for 1 M NaCl C_6_-ssDNA samples.

It must be noted that for ultrathin films, as the monolayers analyzed in this study, refractive index and thickness are highly correlated parameters. A convenient approach to disentangle this correlation is to couple SE with another experimental method. To this end, we previously coupled SE analysis with Electrochemical Impedance Spectroscopy to investigate alkanethiol and protein monolayers [61]. In the present study, nanoshaving experiments have been advantageously exploited to disentangle this correlation: assuming the SAM thickness values obtained from AFM, it is possible to obtain an estimation of the refractive index through a comparison between ellipsometric data and simulated curves, following the approach described in Pinto et al. [45].

Limiting the analysis to the biofilm range of transparency (above 650 nm) and setting the film thickness, we calculated difference spectra using a 4-layer model (ambient | layer | interface | substrate). The analysis of the whole SE spectra of 1 M NaCl C_6_-ssDNA films will be considered in a dedicated paper, where we will focus on the absorption features. In the near-IR region, the DNA film and the ambient were modeled as transparent layers using the Cauchy equation (n(λ) = A + B/$\lambda^{2}$ + C/$\lambda^{4}$) to simulate the dispersion of the refractive index. The coefficient A represents the NIR value of n, while B and C account for dispersion. As concerns the ambient, starting from Krivacic results [62] and taking into account the salt concentration [63], we chose A = 1.33, B = 0.003 µm^2^, and C = 0.00005 µm^4^ for the 1 M NaCl buffer, and A = 1.32, B = 0.003 µm^2^, C = 0.00003 µm^4^ for the 1 mM NaCl buffer. C = 0 µm^4^ was chosen to simulate the DNA layer. Interface effects were included via a Bruggeman Effective Medium Approximation (BEMA) layer, which mixes the dielectric functions of substrate and Cauchy layer with suitable weights [64], exploiting the relative fractions of the two mixing components (i.e., f_Cauchy_ + f_Au_ = 1). This interface layer allows simulating the negative δΨ values in the NIR region. The Au optical constants, which showed good agreement with literature, were obtained by inversion of the spectra of bare substrates [65], as done in previous papers [58].

**Figure 5:**
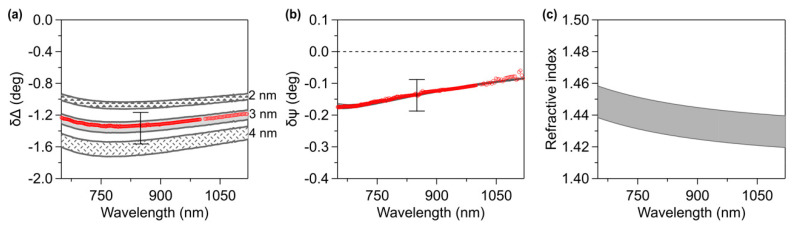
(**a,b**) Comparison between NIR SE δ-data for C_6_-ssDNA prepared in 1 M NaCl buffer (red circles) and simulations (grey lines). Areas decorated with different motifs represent, for d_film_ = 2 nm, 3 nm and 4 nm, simulations with Cauchy A-coefficient values comprised between 1.41 (top border) and 1.43 (bottom border), B = 0.012 µm^2^. Error bars take into account the sample to sample variability. (**c**) Refractive index referred to simulations with d_film_ = 3 nm, B = 0.012 µm^2^ and A-coefficient values comprised between 1.43 (top border) and 1.41 (bottom border).

In Figure 5a and in Figure 5b, data referring to C_6_-ssDNA SAMs prepared in 1 M NaCl buffer are compared with difference spectra calculated by setting the thickness of the film (d_film_ = d_Cauchy_ + d_EMA_ × f_Cauchy_). The shaded areas represent Cauchy simulations with A-coefficient values comprised between 1.41 (top border) and 1.43 (bottom border) (B = 0.012 µm^2^). Guided by the AFM results, we set the film thickness to 3 nm, obtaining the middle curves. Curves calculated with the same A and B values and thicknesses equal to 2 nm and 4 nm are shown for useful comparison (upper and lower shaded regions, respectively). The curves have been calculated using a BEMA layer with a thickness (d_EMA_) of 0.35 nm and a fraction of the Cauchy layer (f_Cauchy_) of 75%. The use of shading to present the δΔ calculated curves provides a graphical visualization of the thickness/refractive index correlation, which characterizes ultrathin films (Figure 5a). On the other hand, calculated δΨ curves are almost superimposed, indicating a very low dependence of δΨ on the refractive index and thickness in the NIR region (Figure 5b).

It can be observed that δΔ and δΨ experimental data results can be superimposed on the calculated curves obtained well, setting the film thickness to the AFM result (3 nm) and using an A coefficient ranging from 1.41 to 1.43. The refractive index used to simulate the Cauchy layer representing the DNA film is shown in Figure 5c. The estimation of the refractive index, around 1.45 at λ = 650 nm, is in good agreement with previous works based on similar systems [66,67,68]; the value is also reasonable comparing with values assumed in experiments on dry DNA thin films [69,70]. In other reports [17,71,72,73], refractive index values comprised between 1.45 and 1.58 were assumed, based on typical values reported for alkanethiols SAMs. In particular, a refractive index of about 1.45 is generally associated to a high film density [53,74,75], confirming the compactness of 1 M C_6_-ssDNA SAMs.

We note that in Pinto et al. [45], SE spectra, accidentally referred to as 1 M NaCl data, were actually 1 mM NaCl data. Therefore, those spectra must not be directly compared with the ones presented in this paper. As discussed in [45], the analysis of subnanometer thick films is critical, since building up an optical model for such thin layers to disentangle the refractive index and thickness can may lead to physically meaningless results. For this reason, in the present work, we focus on the analysis of 1 M NaCl C_6_-ssDNA films and discard the analysis of 1 mM NaCl C_6_-ssDNA which, as shown if Figure 4, is characterized by a very small optical thickness.

## 4. Conclusions

We investigated the role of ionic strength on the morphological and mechanical properties of thiolated DNA SAMs on flat gold substrates. By hard tapping AFM nanoshaving, molecules have been removed from selected areas. QI mode has been introduced, in parallel to the more commonly used soft contact mode, to image shaved areas. The comparative analysis of the AFM results obtained with the two imaging modes indicates that C_6_-ssDNA SAMs deposited at high ionic strength are thicker, stiffer, and less prone to deformation with respect to C_6_-ssDNA SAMs deposited at low ionic strength. This finding is in agreement with the XPS observation of more intense molecular signals, i.e., higher coverage, on films prepared at high salt concentration. Further confirmation comes from the SE data, which provided, through a combined SE/AFM analysis, an estimation of the SAM refractive index in the NIR region, around 1.45, which is a typical value for compact organic films. Remarkably, on the thicker films, SE data allowed clearly detecting DNA molecular absorption at the monolayer level.

The combined AFM, SE, and XPS approach can be further exploited to investigate surface-confined molecular recognition events: in particular, we plan to use DNA SAMs deposited at high ionic strength as suitable platforms to identify specific viral DNA/RNA sequences through detection of helix–helix hybridization.

## Supplementary Materials

The following are available online at https://www.mdpi.com/1996-1944/13/13/2888/s1, Figure S1: AFM force-distance extending curve, Figure S2: XPS survey spectra, Figure S3: high resolution XPS spectra of P2p and N1s core level regions.
