# Supplementary material for: Morphological and Mechanical Characterization of DNA SAMs Combining Nanolithography with AFM and Optical Methods
Source: Materials (Basel). 2020 Jun 27;13(13):2888. doi: 10.3390/ma13132888 (PMC7372444; doi:10.3390/ma13132888)
Supplement: Supplementary file 1 [file materials-13-02888-s001.pdf]

materials

Supporting information

Morphological and Mechanical Characterization of DNA SAMs Combining Nanolithography with AFM and Optical Methods

Giulia Pinto [1,2], Paolo Canepa [1,2], Claudio Canale [1,*], Maurizio Canepa [1,2] and Ornella Cavalleri [1,2,*]

1 Department of Physics, University of Genova, via Dodecaneso 33, 16146, Genova, Italy; pinto@fisica.unige.it (G.P.); paolo.canepa@edu.unige.it (P.C.); canepa@fisica.unige.it (M.C.)
2 OPTMATLAB, Department of Physics, University of Genova, via Dodecaneso 33, 16146, Genova, Italy
* Correspondence: canale@fisica.unige.it (C.C.); cavalleri@fisica.unige.it (O.C.); Tel.: +39 010 3536257 (C.C.); +39 010 3536087 (O.C.)

Received: 24 May 2020; Accepted: 25 June 2020; Published: 27 June 2020

AFM Force Curve

Figure S1 reports a typical experimental force-distance curve acquired in QI mode on a 1 M C_6-ssDNA sample.

To obtain the slope images, for each force curve a linear fit has been adapted to the extending curve in the contact region (black circle). The slope of the linear fit has been used to reconstruct the slope image.

The Young's modulus of the sample was calculated applying the Hertz model [1] to the contact region of the extending curve starting from the contact point. A parabolic shape for the tip (radius: 10 nm) was considered. The original Hertz model was an approximation for the contact of two spheres but nowadays the term is usually used to refer to a family of different models that have been adapted for simple indentation geometries [2,3].

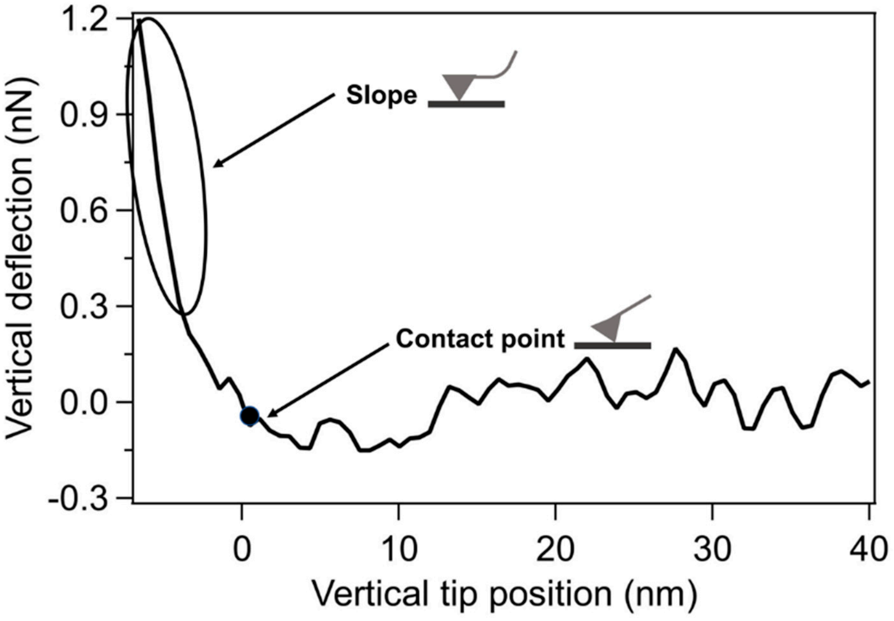

Figure S1. Example of extending trace in a force-distance curve acquired in QI mode.

X-ray Photoelectron Spectroscopy (XPS)

The chemical analysis of the sample surfaces was carried out by XPS measurements using a PHI 5600 Multi-Technique apparatus, with an X-ray Al-monochromatized source (hν = 1486.6 eV). The photoelectron take-off angle was set to 45 degrees. Survey spectra were acquired using a pass-energy of 187.85 eV, while high resolution spectra were acquired with a pass-energy of 23.5 eV. XPS spectra were analysed with Casa-XPS software. A linear background was subtracted from raw data. For the $Au4f_{7/2}$ signal, the Doniach-Sunjic model [4] was used to model the asymmetric metallic behaviour. For the other elements, Voigt functions (30% Gaussian) were used for signal deconvolution. Spin-orbit splitting values used for deconvolution were 0.86 eV for P2p and 3.7 eV for $Au4f_{7/2}/f_{5/2}$. The binding energy scale was calibrated by setting the $Au4f_{7/2}$ peak at 84.0 eV.

Figure S2 reports a typical survey spectrum acquired on a 1 M NaCl C_6-ssDNA SAM which shows the presence of the characteristic molecular elements (sulfur signal is not visible due to the very low signal/noise ratio – there is only a sulfur atom per DNA strand).

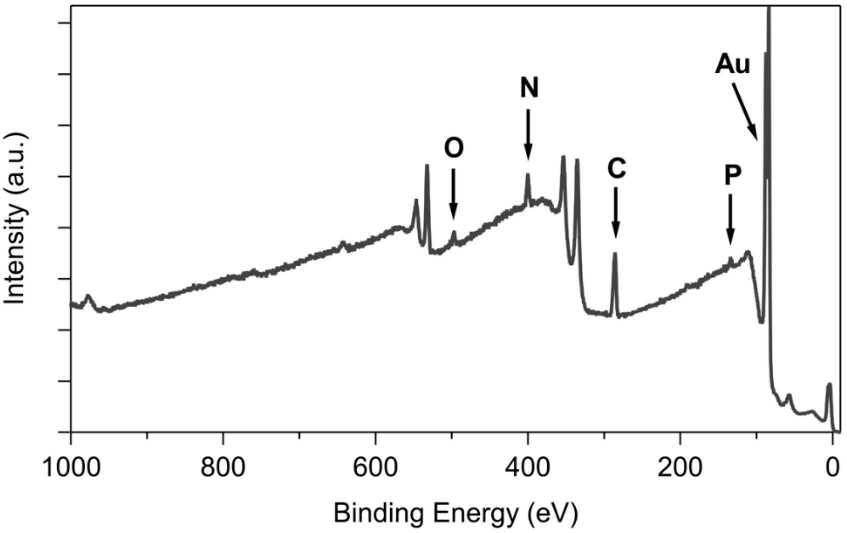

Figure S2. XPS survey referred to C_6-ssDNA SAMs prepared in 1 M NaCl buffer.

High resolution spectra of the P2p and N1s core level regions acquired on 1 M NaCl C_6-ssDNA SAMs and 1 mM NaCl C_6-ssDNA SAMs are reported in Figure S3a,b and Figure S3c,d, respectively.

The P2p core level region (Figure S3a,c) has been deconvoluted with a single doublet with the main 2p3/2 component at (133.8 ± 0.2) eV for 1 M NaCl C_6-ssDNA SAMs and (133.6 ± 0.2) eV for 1 mM NaCl C_6-ssDNA SAMs. The P2p signal can be assigned to the phosphate groups of the oligonucleotide backbone [5–7].

The N1s signal of SAMs prepared at high ionic strength (Figure S3b) has been deconvoluted with two components: N1, at a BE of (399.6 ± 0.2) eV (orange curve), which contains the contribution of N atoms involved in double bonds with C (N=C), and N2, at a BE of (401.1 ± 0.2) eV (green curve), which contains the contribution of N atoms involved in single bonds with C and H atoms [8–11]. Similarly to the spectrum in panel b, the N1s signal of SAMs prepared al low ionic strength (Figure S3d) has been deconvoluted with two components, N1 at (399.4 ± 0.2) eV and N2 at (400.5 ± 0.2) eV.

It can be observed that the signal/noise ratios in 1 mM NaCl data are definitely lower than the 1 M NaCl ones: the lower intensity of the molecular related signals is due to the lower molecular coverage of these films, which can be ascribed to the electrostatic repulsion between negatively charged DNA backbones. In fact, the electrostatic repulsion is reduced at high ionic strength and higher coverage monolayers can form. Similar results have been obtained from Herne and Tarlov [12] in 1 M KH_2PO_4 buffer.

The signal/noise ratios are lower for 1 mM NaCl C_6-ssDNA SAMs also for the other molecular relevant signals (C1s, O1s, S2p). In particular, the ratio between the total molecular signal intensities

and the gold signal intensity is about three times larger in the case of 1 M NaCl films with respect to 1mM NaCl films.

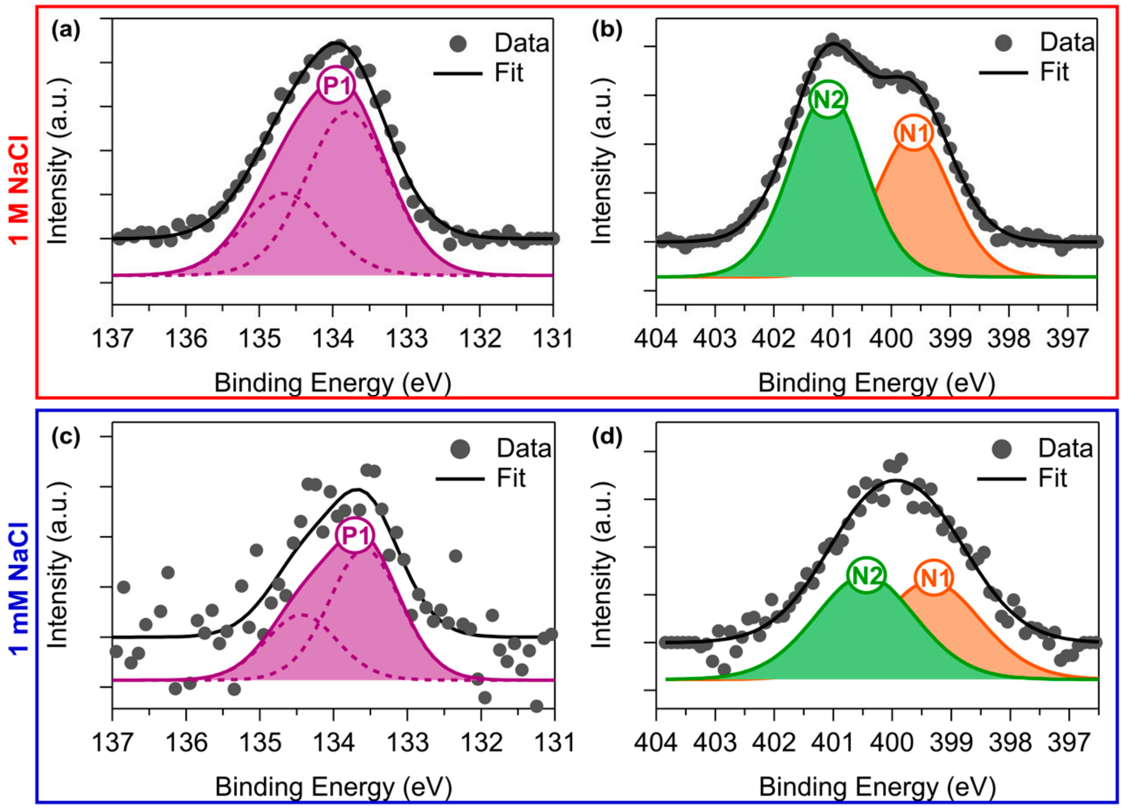

Figure S3. High resolution XPS spectra referred to C_6-ssDNA SAMs prepared in (**a**,**b**) 1 M NaCl and (**c**,**d**) 1 mM NaCl buffer: (**a**,**c**): P2p spectra; (**b**,**d**): N1s spectra.

References

1. Hertz, H. Über die Berührung fester elastischer Körper. *J. Angew. Math.* **1881**, *92*, 156–171.
2. Johnson, K.L. *Contact Mechanics*; Cambridge University Press: Cambridge, UK, 1985; ISBN 0-521-34796-3.
3. Rico, F.; Roca-Cusachs, P.; Gavara, N.; Farré, R.; Rotger, M.; Navajas, D. Probing mechanical properties of living cells by atomic force microscopy with blunted pyramidal cantilever tips. *Phys. Rev. E* **2005**, *72*, 021914, doi:10.1103/PhysRevE.72.021914.
4. Doniach, S.; Sunjic, M. Many-electron singularity in X-ray photoemission and X-ray line spectra from metals. *J. Phys. C: Solid State Phys.* **1970**, *3*, 285–291, doi:10.1088/0022-3719/3/2/010.
5. Petrovykh, D.Y.; Kimura-Suda, H.; Tarlov, M.J.; Whitman, L.J. Quantitative Characterization of DNA Films by X-ray Photoelectron Spectroscopy. *Langmuir* **2004**, *20*, 429–440, doi:10.1021/la034944o.
6. Lee, C.-Y.; Gong, P.; Harbers, G.M.; Grainger, D.W.; Castner, D.G.; Gamble, L.J. Surface Coverage and Structure of Mixed DNA/Alkylthiol Monolayers on Gold: Characterization by XPS, NEXAFS, and Fluorescence Intensity Measurements. *Anal. Chem.* **2006**, *78*, 3316–3325, doi:10.1021/ac052137j.
7. Howell, C.; Zhao, J.; Koelsch, P.; Zharnikov, M. Hybridization in ssDNA films—A multi-technique spectroscopy study. *Phys. Chem. Chem. Phys.* **2011**, *13*, 15512–15522, doi:10.1039/c1cp20374f.
8. Saprigin, A.V.; Thomas, C.W.; Dulcey, C.S.; Patterson, C.H.; Spector, M.S. Spectroscopic quantification of covalently immobilized oligonucleotides. *Surf. Interface Anal.* **2005**, *37*, 24–32, doi:10.1002/sia.1999.
9. Vilar, M.R.; Botelho do Rego, A.M.; Ferraria, A.M.; Jugnet, Y.; Noguès, C.; Peled, D.; Naaman, R. Interaction of Self-Assembled Monolayers of DNA with Electrons: HREELS and XPS Studies. *J. Phys. Chem. B* **2008**, *112*, 6957–6964, doi:10.1021/jp8008207.
10. Khan, M.N.; Tjong, V.; Chilkoti, A.; Zharnikov, M. Spectroscopic Study of a DNA Brush Synthesized in Situ by Surface Initiated Enzymatic Polymerization. *J. Phys. Chem. B* **2013**, *117*, 9929–9938, doi:10.1021/jp404774x.
11. Bartl, J.D.; Scarbolo, P.; Brandalise, D.; Stutzmann, M.; Tornow, M.; Selmi, L.; Cattani-Scholz, A. Role of

Different Receptor-Surface Binding Modes in the Morphological and Electrochemical Properties of Peptide-Nucleic-Acid-Based Sensing Platforms. *Langmuir* **2019**, *35*, 3272–3283, doi:10.1021/acs.langmuir.8b03968.

12. Herne, T.M.; Tarlov, M.J. Characterization of DNA Probes Immobilized on Gold Surfaces. *J. Am. Chem. Soc.* **1997**, *119*, 8916–8920, doi:10.1021/ja9719586.

© 2020 by the authors. Licensee MDPI, Basel, Switzerland. This article is an open access article distributed under the terms and conditions of the Creative Commons Attribution (CC BY) license (http://creativecommons.org/licenses/by/4.0/).
